# The gut microenvironment in antibody-mediated rejection: from conventional immunosuppression to precision immunomodulation in organ transplantation

**DOI:** 10.3389/fimmu.2026.1968780

**Published:** 2026-10-08

**Authors:** Yoshiko Matsuda

**Affiliations:** Bio Ameka, Osaka, Japan

**Keywords:** antibody-mediated rejection, biomarkers, gut microbiota, immune modulation, organ transplantation

## Abstract

The gut microbiota and broader gut microenvironment, comprising microbiota-derived metabolites and host–microbial interactions, have emerged as potentially modifiable factors associated with host immune regulation and have been implicated in altered drug responsiveness, immune-mediated diseases, and metabolic disorders. Accumulating evidence suggests that the gut microenvironment may modulate post-transplant immune responses and may provide candidate biomarkers and therapeutic targets in organ transplantation. Gut dysbiosis has been associated with immune dysregulation and adverse allograft outcomes, although direct causal evidence in human AMR remains limited. Profiling the gut microenvironment may therefore complement conventional immune monitoring for post-transplant risk stratification. In this review, current evidence on the interactions among the gut microbiota, microbiota-derived metabolites, and host immune regulation is synthesized, with particular emphasis on the role of the gut microenvironment in maintaining immune homeostasis and influencing transplant outcomes. The impact of alterations in the gut microenvironment on the development of antibody-mediated rejection—a major barrier to long-term graft survival—is examined by focusing on their effects on regulatory immune pathways and inflammatory responses. The potential role of microbiota-derived metabolites in promoting immune tolerance and graft accommodation, two complementary mechanisms that contribute to long-term allograft protection, is also discussed. Finally, gut microenvironment-based strategies for early diagnosis, risk stratification, and rejection prediction are evaluated, alongside therapeutic interventions including prebiotics, probiotics, synbiotics, postbiotics, and fecal microbiota transplantation. These strategies may facilitate pre-emptive intervention before irreversible graft injury develops and support a shift from conventional immunosuppression towards precision immunomodulation in transplantation medicine.

## 1 Introduction

Organ transplantation is an established treatment for end-stage organ failure, and advances in immunosuppressive therapy have markedly improved short-term graft outcomes. However, long-term graft survival remains suboptimal, with antibody-mediated rejection (AMR) continuing to represent a major immunological barrier to durable allograft survival (1–5). AMR is characterised by donor-specific human leukocyte antigen (HLA) antibodies (DSAs), complement activation, microvascular inflammation, and endothelial injury, which ultimately lead to irreversible allograft damage (2, 4–7). Successful management of AMR depends on early detection of immunological alterations before overt graft injury occurs, as therapeutic interventions are often less effective once the disease becomes clinically apparent, thereby necessitating pre-emptive intervention (1–5, 8).

Current diagnostic approaches, including serum creatinine, proteinuria, DSA testing, and allograft biopsy, remain essential for post-transplant assessment. However, these methods have limited ability to accurately predict AMR risk, assess disease activity, or forecast long-term graft outcomes. Novel, non-invasive immune-monitoring strategies are therefore needed to dynamically assess post-transplant immune status (9–13).

In this context, the gut microbiota is suggested to modulate host immunity through systemic immune responses through interactions with regulatory T cells (Tregs), T helper (Th)17 cells, dendritic cells (DCs), B cells, and macrophages. Microbiota-derived metabolites—particularly SCFAs—may contribute to intestinal barrier integrity and immune regulation, while their potential roles in transplant tolerance and graft accommodation remain under investigation (14–25). Following organ transplantation, the composition and function of the gut microbiota may be altered by immunosuppressive drugs, antimicrobial agents, infections, and organ dysfunction, resulting in dysbiosis that may influence allograft rejection, susceptibility to infection, metabolic complications, and responses to immunosuppressive treatment (14, 26–29).

Accordingly, analysis of the gut microenvironment may offer a novel approach to immune monitoring after transplantation. Beyond characterising microbial composition and diversity, integrating functional information from microbiota-derived metabolites, particularly SCFAs, may improve risk stratification and facilitate earlier detection of post-transplant complications, including AMR (9, 12, 26, 28, 30). As the gut microbiota is also a modifiable therapeutic target, it presents opportunities for immune monitoring and early diagnosis as well as for pre-emptive intervention (14, 26, 28, 31–35). Recent advances in artificial intelligence (AI) and multi-omics approaches have further expanded the potential of gut microenvironment-based approaches. By integrating microbiome composition, microbial metabolites, host immune signatures, and clinical variables, AI-driven predictive models may enable earlier identification of patients at risk of AMR by correlating metagenomic dysbiosis scores (e.g., Faecalibacterium depletion) and plasma SCFA levels in relation to activation of humoral immune responses against the graft and graft transcriptomics to guide personalized immunomodulatory strategies in transplantation (1–10, 14–21, 26–30).

This review summarises current evidence linking the gut microbiota and microbiota-derived metabolites to transplant immunity, with particular emphasis on their role in the pathogenesis of AMR. The potential of gut microenvironment-based approaches for immune monitoring, early risk stratification, and the development of preventive and therapeutic strategies aimed at improving long-term allograft outcomes is also explored.

## 2 Molecular pathophysiology of the gut microenvironment in organ transplantation

The fate of a transplanted organ is determined by the dynamic balance between immune responses that promote allograft rejection and regulatory mechanisms that maintain immune tolerance and graft accommodation. Increasing evidence suggests that the gut microenvironment may influence this balance (14, 26, 36, 37). The gut microenvironment comprises the gut microbiota, intestinal epithelial barrier, gut-associated lymphoid tissue, immune cells, and microbiota-derived metabolites, all of which influence local and systemic immune responses (14, 16–20). Although immunosuppressive agents are indispensable for preventing rejection, they can also alter the composition and function of the gut microbiota. The gut microenvironment may therefore contribute to variation in transplant immunity and allograft outcomes (14, 26–29).

### 2.1 Gut microenvironment in allograft rejection

#### 2.1.1 Antibody-mediated rejection

AMR is a major cause of allograft dysfunction and is primarily driven by humoral immune responses mediated by B cells and plasma cells (PCs) (1–8). DSAs, the principal pathogenic antibodies in AMR, are generated following the presentation of donor-derived HLA molecules to recipient CD4+ T cells by antigen-presenting cells. Activated CD4+ T cells differentiate into T follicular helper (Tfh) cells, which promote B-cell activation, germinal centre formation, class-switch recombination, and affinity maturation. Subsequently, B cells differentiate into memory B cells (mBCs) and long-lived plasma cells (LLPCs), which continuously produce DSAs. Binding of DSAs to donor HLA molecules expressed on graft endothelial cells activates the classical complement pathway, leading to C1q binding, C4d deposition, and membrane attack complex formation. These events induce endothelial injury, microvascular inflammation, thrombosis, and progressive graft dysfunction, ultimately resulting in graft failure (1–8, 38) (**Figure 1**).

**Figure 1:**
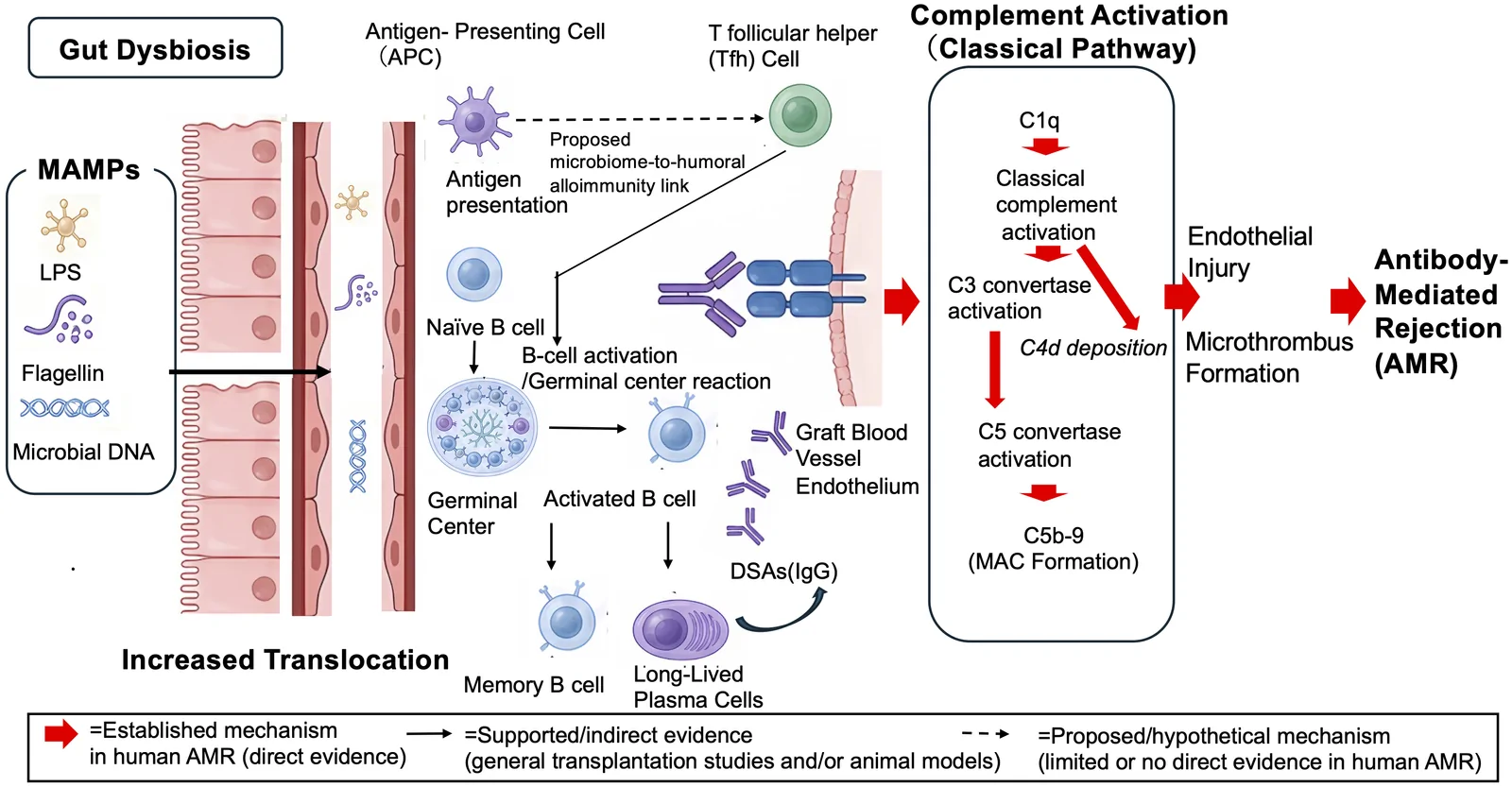
Gut dysbiosis and the pathogenesis of antibody-mediated rejection (AMR). Post-transplant clinical interventions, particularly the concomitant use of immunosuppressants and antibiotics, frequently alter the recipient’s gut microbial ecosystem. This shift is characterized by a profound loss of taxonomic diversity and a marked decline in short-chain fatty acid (SCFA)-producing genera, which impairs the integrity of the intestinal epithelial barrier and allows the systemic translocation of microbe-associated molecular patterns (MAMPs) such as lipopolysaccharide (LPS) (14, 16, 26, 28, 56). Once in circulation, these microbial products engage Toll-like receptors (TLRs) on innate immune cells, promoting pro-inflammatory cytokines—including tumor necrosis factor-α, interleukin-6 (IL-6), and interleukin-1β (IL-1β)—and driving downstream adaptive immune activation (9, 14, 26, 28, 38). This sustained inflammatory milieu is hypothesized to enhance T follicular helper (Tfh) cell-mediated germinal center reactions, B-cell maturation, and subsequent differentiation into donor-specific HLA antibody (DSA)-producing PCs (4, 5, 38, 45, 46). This pathogenic link is further supported by newly emerging clinical data showing that specific pre-transplant dysbiosis profiles—characterized by the expansion of Proteobacteria and the loss of immunoregulatory taxa—independently correlate with a heightened risk of early humoral activation and subsequent rejection episodes (88). Binding of circulating DSAs to donor HLA molecules on the graft endothelium can activate the classical complement pathway through C1q binding, leading to C4d deposition, C3 and C5 convertase activation, and subsequent C5b-9 membrane attack complex (MAC) formation, thereby contributing to endothelial injury, microvascular inflammation, and microthrombus formation in AMR (4, 5, 38). AMR, antibody-mediated rejection; Breg, regulatory B cell; C1q, complement component 1q; C4d, complement component 4d; DC, dendritic cell; DSA, donor-specific HLA antibody; HLA, human leukocyte antigen; IL-6, interleukin-6; IL-1β, interleukin-1 beta; LPS, lipopolysaccharide; MAC, membrane attack complex; MAMP, microbe-associated molecular pattern; RBC, red blood cell; SCFA, short-chain fatty acid; Tfh, T follicular helper cell; TLR, Toll-like receptor; TNF-α, tumor necrosis factor-α; Treg, regulatory T cell.

#### 2.1.2 T cell-mediated rejection

T cell-mediated rejection (TCMR) is predominantly driven by cellular immune responses. Donor antigens are recognised through direct, indirect, and semi-direct allorecognition pathways. Following antigen recognition, CD4+ T cells differentiate into Th1 and Th17 cells. Th1 cells produce interferon -γ and tumour necrosis factor-α, which activate macrophages and amplify inflammatory responses, whereas Th17 cells secrete interleukin (IL)-17 and IL-22, promoting neutrophil recruitment and chronic inflammation. In parallel, CD8+ cytotoxic T cells directly damage graft cells through perforin/granzyme B- and Fas/Fas ligand-mediated cytotoxicity. These immune responses result in tubulitis, endarteritis, and interstitial inflammation, the characteristic pathological features of TCMR (21, 39–44) (**Figure 2**).

**Figure 2:**
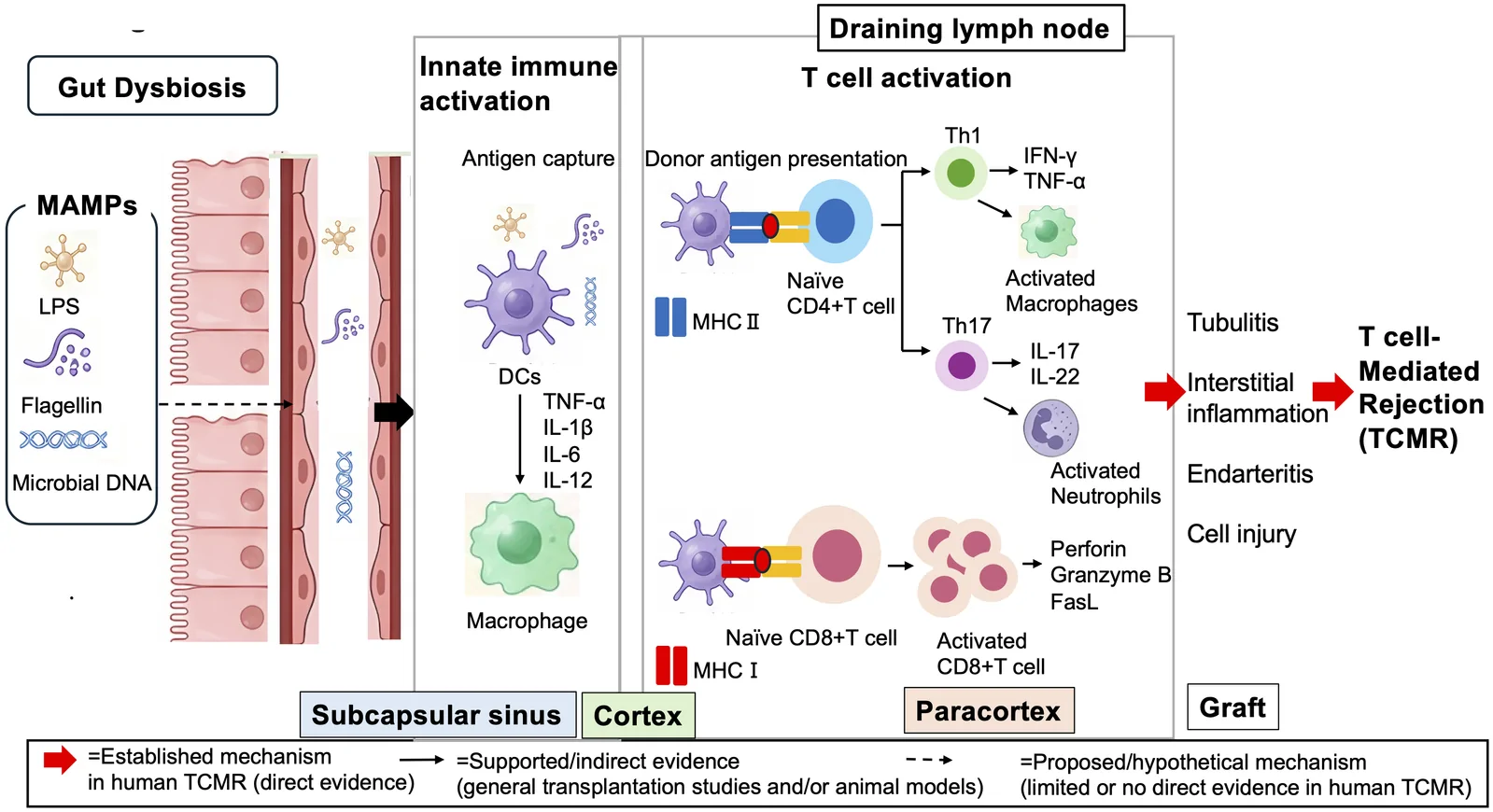
Pathways linking intestinal microbial shifts to T cell-mediated rejection (TCMR) within the specific anatomical compartments of the lymph node. Disruption of the intestinal microenvironment by transplant-related medications compromises the integrity of the gut barrier, facilitating the systemic translocation of lumen-derived microbe-associated molecular patterns (MAMPs) such as lipopolysaccharide (LPS) and peptidoglycan (14, 18–21, 26–28). These circulating microbial signals stimulate dendritic cells (DCs) and macrophages via Toll-like receptor (TLR) pathways, establishing a pro-inflammatory cytokine profile rich in tumor necrosis factor-α (TNF-α), interleukin-1β (IL-1β), interleukin-6 (IL-6), and interleukin-12 (IL-12) within the subcapsular sinus (14, 17, 20, 21, 26–28). In parallel, donor alloantigens are presented to recipient T cells within the lymph node cortex. In this cortical zone, recipient naïve CD4+ and CD8+ T cells engage donor antigens via MHC class II and MHC class I molecules, respectively, through direct, indirect, and semi-direct allorecognition pathways. Under the influence of this dysbiosis-induced inflammatory environment, these pathways selectively drive the polarization of CD4+ T cells into T helper 1 (Th1) and T helper 17 (Th17) phenotypes within the paracortex, while simultaneously triggering the clonal expansion of CD8+ cytotoxic T cells (21, 39–44). Effector cytokines derived from Th1 cells (interferon-γ [IFN-γ] and TNF-α) activate macrophages to amplify tissue inflammation, whereas Th17-derived interleukin-17 (IL-17) and interleukin-22 (IL-22) promote robust neutrophil infiltration toward the medulla (14, 17, 20, 21). Ultimately, activated CD8+ T cells exert targeted allograft injury via perforin/granzyme B and Fas/Fas ligand (Fas/FasL) pathways, manifesting histopathologically as tubulitis, interstitial inflammation, and endarteritis during TCMR development (21, 39–44). CD4+, cluster of differentiation 4; CD8+, cluster of differentiation 8; DC, dendritic cell; Fas/FasL, Fas cell surface death receptor/Fas ligand; IFN-γ, interferon-gamma; IL-6, interleukin-6; IL-12, interleukin-12; IL-17, interleukin-17; IL-22, interleukin-22; IL-1β, interleukin-1 beta; LPS, lipopolysaccharide; MAMP, microbe-associated molecular pattern; TCMR, T cell-mediated rejection; Th1, T helper 1 cell; Th17, T helper 17 cell; TLR, Toll-like receptor; TNF-α, tumor necrosis factor-α.

#### 2.1.3 Comparison between antibody-mediated rejection and T cell-mediated rejection

Although AMR and TCMR are traditionally regarded as distinct forms of allograft rejection, they share several pathogenic mechanisms. Both AMR and TCMR are initiated by recognition of donor-derived antigens and are influenced by innate immune activation, antigen-presenting cells, pro-inflammatory cytokines, and the balance between effector and regulatory immune responses. Gut dysbiosis potentially contributes to both AMR and TCMR through the disruption of intestinal barrier integrity, systemic inflammation, and the downstream activation of alloimmune pathways (14, 17, 26, 39–44).

Despite these shared mechanisms, important differences distinguish the two processes. TCMR is primarily mediated by cellular immune responses involving Th1 cells, Th17 cells, and cytotoxic T lymphocytes, whereas AMR is driven predominantly by humoral immunity, including Tfh-cell-dependent B-cell activation, DSA production, complement activation, and endothelial injury (1–8, 38–44). Clinically, TCMR is generally more responsive to conventional immunosuppressive therapies, whereas AMR remains more difficult to predict, prevent, and treat, particularly after the establishment of LLPCs and persistent DSA production (1–8, 39–44).

Hence, this review focuses primarily on AMR. Although advances in immunosuppressive therapy have substantially reduced the incidence of acute TCMR, AMR remains one of the principal barriers to long-term allograft survival. The gut microenvironment may influence processes relevant to AMR pathogenesis, including DSA generation and immune regulation, while its potential relationship with graft accommodation remains to be established. A better understanding of these interactions may facilitate preclinical prediction, improve risk stratification, and support the development of precision immunomodulatory strategies in organ transplantation (1–8, 14, 26, 28, 38).

#### 2.1.4 Impact of gut dysbiosis on allograft rejection

Under physiological conditions, the gut microbiota maintains intestinal barrier integrity and supports immune homeostasis (14, 18–20, 26). However, immunosuppressive agents, antibiotics, and other transplant-related factors may induce dysbiosis, increasing intestinal permeability and potentially facilitating the translocation of microbe-associated molecular patterns, including lipopolysaccharide and peptidoglycan, into the systemic circulation (14, 19, 26–28). In generalized transplantation models, these translocated microbial products engage Toll-like receptors (TLRs) on dendritic cells and macrophages, subsequently stimulating the baseline production of pro-inflammatory cytokines and modulating systemic adaptive immune reactivities (9, 14, 26, 28, 38). Crucially, in the specific pathogenesis of AMR, these circulating MAMPs are hypothesized to provide a potent innate stimulus that accelerates the humoral alloimmune arm. Specifically, these products stimulate TLRs on antigen-presenting cells to augment the T follicular helper (Tfh)–germinal center axis, thereby promoting B-cell activation, somatic hypermutation, and subsequent differentiation into donor-specific HLA antibody (DSA)-producing plasma cells (4, 5, 38, 45, 46). Binding of circulating DSAs to donor HLA molecules on the graft endothelium can activate the classical complement pathway through C1q binding, leading to C4d deposition, C3 and C5 convertase activation, and subsequent C5b-9 membrane attack complex (MAC) formation, thereby contributing to endothelial injury, microvascular inflammation, and microthrombus formation in AMR (4, 5, 38) (**Figure 1**). In contrast, as derived primarily from generalized transplantation models and experimental studies of cellular rejection, this dysbiosis-associated inflammatory milieu alternatively favors Th1- and Th17-cell differentiation, augmenting cytotoxic T-cell responses and accelerating cell-mediated tubulitis and interstitial inflammation during TCMR development (14, 17, 20, 21, 40, 41, 43, 44) (**Figure 2**).

Direct experimental transplantation studies further support a causal role of the gut microbiota in modulating alloimmune responses. In murine transplantation models, microbiota composition influenced allograft rejection by modulating antigen-presenting-cell function and alloreactive T-cell responses (47). In addition, microbiota modulation has been associated with reduced memory CD8+ T-cell responses and prolonged allograft survival (48).

### 2.2 Gut microenvironment in immune tolerance and accommodation

#### 2.2.1 Immune tolerance

Immune tolerance is a fundamental mechanism that suppresses transplant immune responses. It refers to a state in which the allograft is recognised as foreign but does not elicit a destructive immune response and can be broadly classified as central or peripheral tolerance (36, 37). Central tolerance is established in the thymus through deletion of donor-reactive T cells. Under conditions of mixed chimerism, donor-derived haematopoietic cells migrate to the thymus and promote the negative selection of donor-reactive T cells (36, 49, 50). In contrast, peripheral tolerance is primarily mediated by Tregs, which suppress immune responses through the production of IL-10, transforming growth factor-β (TGF-β), and cytotoxic T-lymphocyte-associated protein 4.

Regulatory B cells (Bregs) further contribute to the maintenance of immune tolerance by producing IL-10 and TGF-β, suppressing inflammatory T-cell responses, and promoting Treg differentiation (4, 14, 36, 51–53). Recently, IgM memory B cells (mBCs) have been recognized as a Breg-like population exhibiting immunoregulatory properties (45, 54). These cells may contribute to the maintenance of immune tolerance through IL-10 production and Treg induction, and increased frequencies have been reported in operationally tolerant transplant recipients. These findings support a potential role for these cells in long-term immune homeostasis (4, 12, 36, 45, 53–55). Microbiota-derived metabolites support regulatory immune networks involving Tregs and Bregs, as well as IgM mBC maintenance, supporting a potential role for the gut microenvironment in transplant tolerance (14, 17, 36, 45, 53–55).

Accumulating evidence suggests that the gut microbiota may serve as a modulator of immune tolerance. Preclinical models indicate that SCFAs, particularly butyrate produced by Faecalibacterium prausnitzii and members of Clostridium clusters IV and XIVa, can promote forkhead box P3 (FoxP3) expression through histone deacetylase inhibition, thereby supporting Treg differentiation (14, 17, 56, 57). Experimental data also indicate that SCFAs may enhance Breg induction and IL-10 production, creating an immunoregulatory environment that favours transplant tolerance (14, 17, 45, 55) (**Figure 3**). Furthermore, recent translational perspectives have highlighted the potential relevance of regulatory T- and B-cell networks to the maintenance of allograft tolerance (58). Importantly, these immunoregulatory concepts are supported by direct experimental transplantation studies. In murine cardiac transplantation, microbiota manipulation altered allograft outcomes, with enrichment of Bifidobacterium pseudolongum associated with prolonged graft survival and reduced inflammation and fibrosis (59). In murine kidney transplantation, a high-fiber diet or acetate supplementation promoted long-term allograft survival and donor-specific tolerance through Treg- and GPR43-dependent mechanisms (60). These findings provide direct transplantation-specific evidence linking the gut microbiota and microbial metabolites to the regulation of alloimmune responses, although their relevance to human AMR remains to be established.

**Figure 3:**
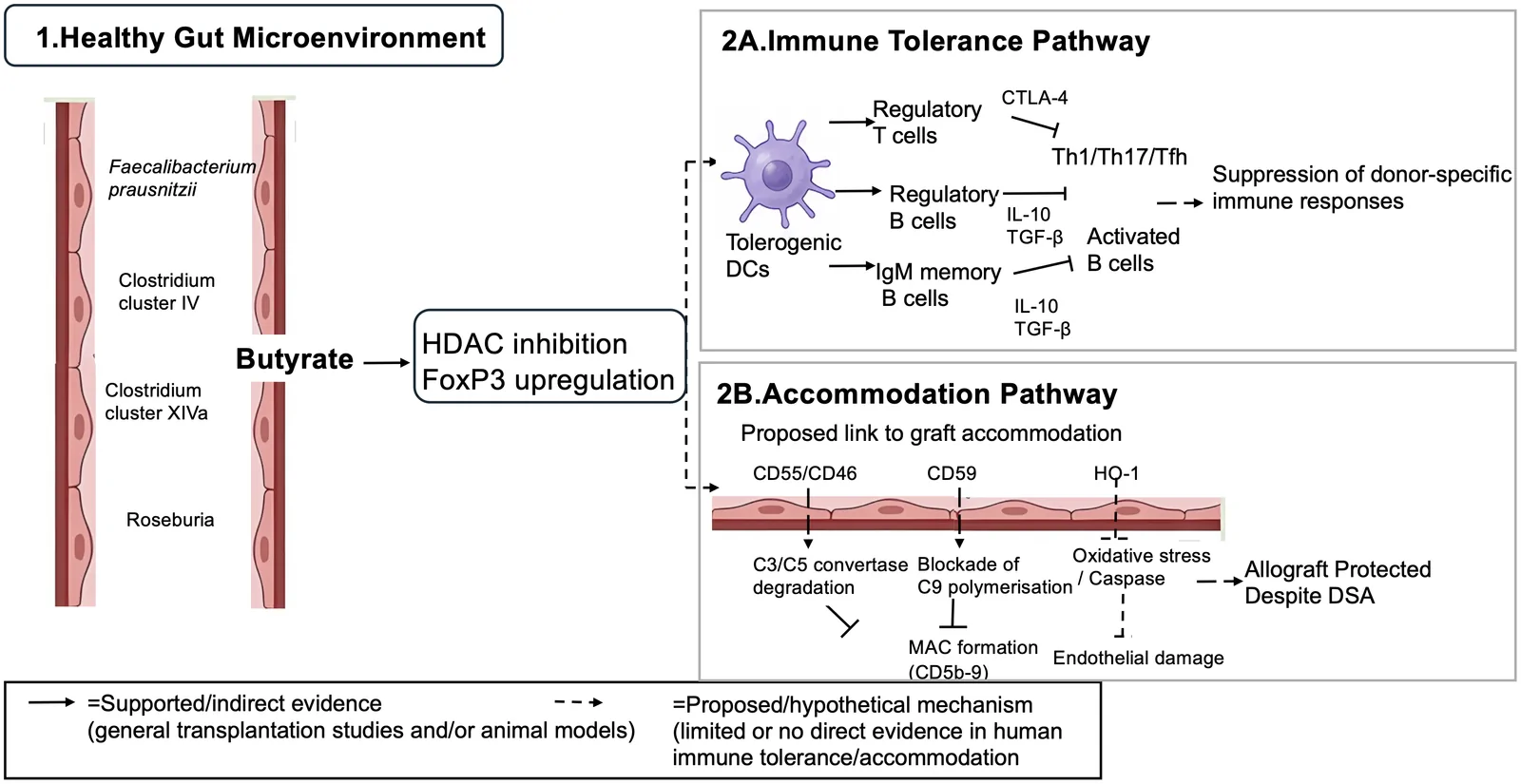
Symbiotic microbial mechanisms supporting transplant tolerance and graft accommodation. A homeostatic gut microbiota preserves intestinal barrier function and sustains a robust output of short-chain fatty acids (SCFAs), notably butyrate synthesized by Faecalibacterium prausnitzii and Clostridium clusters IV and XIVa, and Roseburia (14, 17, 56, 57). **(A)** Immune tolerance: Luminal SCFAs function as histone deacetylase (HDAC) inhibitors to upregulate forkhead box P3 (FoxP3) expression, thereby promoting the differentiation and peripheral expansion of regulatory T cells (Tregs), regulatory B cells (Bregs), and immunoregulatory IgM memory B cells (14, 17, 36, 45, 46, 51–57, 67, 75). These regulatory subsets secrete potent anti-inflammatory mediators (interleukin-10 [IL-10] and transforming growth factor-β [TGF-β]) and express cytotoxic T-lymphocyte-associated protein 4 (CTLA-4), effectively suppressing effector T-cell lineages to maintain systemic immune homeostasis (36, 45, 46, 51–57, 67, 75). **(B)** Accommodation: Microbiota-derived metabolites are also implicated as a proposed regulatory mechanism in establishing allograft accommodation, a protective state wherein the graft resists injury despite persistent donor-specific HLA antibody (DSA) positivity (6, 22, 23, 38). This phenomenon is driven by the upregulation of complement regulatory proteins (CD46, CD55, and CD59) and cytoprotective cascades (B-cell lymphoma 2 [Bcl-2], B-cell lymphoma-extra-large [Bcl-xL], and heme oxygenase-1 [HO-1]) on the graft endothelium, which shield cells from complement-mediated lysis and oxidative stress (6, 24, 25). While these basic cytoprotective pathways are characterized on the graft endothelium, the direct causal role of gut microbiota-derived butyrate in driving clinical human graft accommodation remains hypothetical and requires further clinical validation, as reflected by the proposed arrow designations. Bcl-2, B-cell lymphoma 2; Bcl-xL, B-cell lymphoma-extra-large; Breg, regulatory B cell; CD46, cluster of differentiation 46; CD55, cluster of differentiation 55; CD59, cluster of differentiation 59; CTLA-4, cytotoxic T-lymphocyte-associated protein 4; DSA, Donor-specific HLA antibody; FoxP3, forkhead box P3; HDAC, histone deacetylase; HO-1, heme oxygenase-1; IL-10, interleukin-10; SCFA, short-chain fatty acid; TGF-β, transforming growth factor-beta; Treg, regulatory T cell.

#### 2.2.2 Accommodation

Alongside immune tolerance, accommodation represents another protective mechanism in transplantation. Accommodation refers to a state in which DSAs are present but fail to induce allograft injury and is most commonly observed following ABO-incompatible transplantation (6, 22, 23, 38). Accommodated grafts exhibit increased expression of complement regulatory proteins, including CD46, CD55, and CD59, which protect endothelial cells from complement-mediated injury. They also upregulate anti-apoptotic and cytoprotective molecules, such as B-cell lymphoma 2, B-cell lymphoma-extra-large, and haem oxygenase-1, thereby enhancing resistance to complement activation and oxidative stress-induced cellular injury. These cytoprotective mechanisms thus shield the allograft from injury despite persistent DSAs (6, 24, 25). While direct links between the gut microbiota and graft accommodation are still being defined, microbiota-derived metabolites—particularly SCFAs—have been proposed to support biological processes that could favor graft accommodation, including suppression of excessive inflammation and preservation of endothelial integrity; however, direct evidence that SCFAs induce accommodation in human transplantation is lacking. These immunomodulatory effects may enhance graft resilience despite persistent DSA positivity, indicating that the gut microenvironment could contribute to both immune tolerance and graft accommodation through complementary but distinct mechanisms (14, 17, 22–25, 28, 56, 57) (**Figure 3**).

Overall, post-transplant immune responses are shaped by B-cell and PC pathways responsible for AMR, and T-cell pathways responsible for TCMR, whereas immune tolerance and graft accommodation function as complementary mechanisms that counteract these alloimmune responses. The gut microenvironment may influence several of these processes. Dysbiosis has been associated with allograft rejection, whereas beneficial microbial communities and their metabolites may support regulatory immune pathways. Their potential contribution to graft accommodation and long-term graft survival remains to be established. Although gut dysbiosis has been implicated in both AMR and TCMR, its clinical relevance may be greatest in AMR, which remains a major obstacle to long-term allograft survival (6, 14, 17, 26, 28) (**Figure 3**).

#### 2.2.3 Short-chain fatty acids as regulators of transplant immunity

Beyond microbial composition, microbiota-derived metabolites serve as key functional mediators linking the gut microenvironment to systemic immune regulation (14, 16, 17, 19, 20). Among these, SCFAs, including acetate, propionate, and butyrate, are the best characterised metabolites in transplantation and have been shown to promote regulatory immune responses, as described above.

Transplantation-specific experimental studies further support the immunomodulatory potential of SCFAs. In a murine kidney transplantation model, acetate promoted donor-specific allograft tolerance through GPR43- and Treg-dependent mechanisms (60). More recently, targeted delivery of butyrate to the lower gut prolonged skin allograft survival through modulation of the myeloid compartment (61).

#### 2.2.4 Emerging microbiota-derived metabolites in transplant immunity

Beyond SCFAs, tryptophan-derived indole metabolites represent another key class of immunomodulatory molecules. Produced via bacterial metabolism, these derivatives activate the aryl hydrocarbon receptor (AhR) pathway in intestinal epithelial and immune cells. AhR signalling reinforces epithelial barrier integrity while suppressing hyperinflammation and promoting regulatory cell differentiation. Consequently, impaired tryptophan metabolism and diminished indole generation correlate with compromised immune regulation and heightened inflammatory susceptibility (16, 17, 19). Secondary bile acids also exert important immunomodulatory effects. These metabolites, produced through microbial transformation of primary bile acids in the intestine, also influence immune function by regulating T-cell differentiation, macrophage activation, and cytokine production. Experimental evidence suggests that secondary bile acids may promote regulatory immune responses while suppressing pro-inflammatory pathways, thereby contributing to the maintenance of immune tolerance (16, 17, 19).

These findings suggest that microbiota-derived metabolites may serve as key molecular mediators linking the gut microbiota with host immunity. Alterations in the production of SCFAs, indole derivatives, and secondary bile acids could potentially influence the balance between alloimmune activation and immune regulation following transplantation. Therefore, characterization of metabolite profiles may provide novel biomarkers of transplant immune status and offer mechanistic insights to support the development of strategies that promote immune tolerance, accommodation, and long-term allograft survival (28, 30, 62–65). In summary, these metabolites, their corresponding molecular receptors, and the detailed immunomodulatory pathways underpinning these microbial interventions are comprehensively summarized in **Table 1**.

**Table 1:** Postbiotic metabolites and their immunomodulatory effects on allograft immunity.

Metabolite	Main targets	Key immunomodulatory effects	Potential allograft benefits
SCFAs (acetate, propionate, butyrate)	GPR41, GPR43, GPR109A, HDAC	Promotes Treg differentiation; suppresses Th1/Th17 responses; reduces NF-κB signaling	May reduce allograft inflammation and promote graft tolerance in experimental models
Tryptophan metabolites (indoles, IPA, kynurenine)	AhR	Promotes IL-10/IL-22 production and tolerogenic DCs; suppresses Th17 responses	Enhances gut barrier integrity; reduces systemic alloreactivity
Secondary bile acids (lithocholic acid, deoxycholic acid)	TGR5, FXR, VDR	Suppresses pro-inflammatory cytokines; regulates Th17/Treg balance	Potentially reduces chronic graft vasculopathy; promotes immune homeostasis
Polyamines & microbial peptides (putrescine, spermidine, cell-wall components)	Intracellular signaling; tight-junction proteins	Promotes claudin/occludin expression; reduces oxidative stress and endothelial activation	Reduces ischemia–reperfusion injury; preserves early graft function

## 3 Gut microbiome analysis for the control of antibody-mediated rejection

Building on the mechanistic insights described above, translating the role of the gut microenvironment into clinical practice offers new opportunities to improve AMR risk stratification and management. Rather than duplicating current diagnostic standards, gut microbiome profiling serves as a complementary tool to address the limitations of conventional, invasive approaches. The following sections evaluate the status of current diagnostic and therapeutic strategies for AMR and discuss how gut microbiome analysis can be integrated into actionable clinical workflows to improve long-term transplant outcomes.

Although the gut microenvironment represents a promising therapeutic target, effective strategies for preventing and controlling AMR remain limited, underscoring the need for a deeper understanding of gut-immune interactions and novel interventions (1–7).

The gut microenvironment may modulate alloimmune responses and may influence processes relevant to the onset and progression of AMR. Integrating gut microbiome profiling with conventional pathological and immunological assessments may provide new insights into the mechanisms underlying AMR, facilitate the identification of early diagnostic biomarkers, and support the development of personalized therapeutic interventions (14, 26–28, 30). Accordingly, elucidating the relationship between AMR and gut microenvironment represents an important research priority, with direct implications for improving long-term transplant outcomes.

### 3.1 Challenges in conventional antibody-mediated rejection diagnostics

Diagnosis of AMR currently relies primarily on DSA detection and allograft biopsy (2, 5, 41, 42). However, not all DSAs are pathogenic, and their presence alone is insufficient to accurately assess rejection activity or predict subsequent graft injury (2, 6, 38, 66). Allograft biopsy is inherently invasive, and histologic changes frequently lag behind irreversible tissue injury (2, 9, 13, 41, 42). Existing diagnostic approaches also provide limited insight into the balance between pathogenic humoral immune responses and protective regulatory mechanisms, including immune tolerance and graft accommodation. Effective immune monitoring is thus required to identify patients at risk of AMR prior to clinically apparent graft injury (9–13, 36, 67) (**Figure 4**).

**Figure 4:**
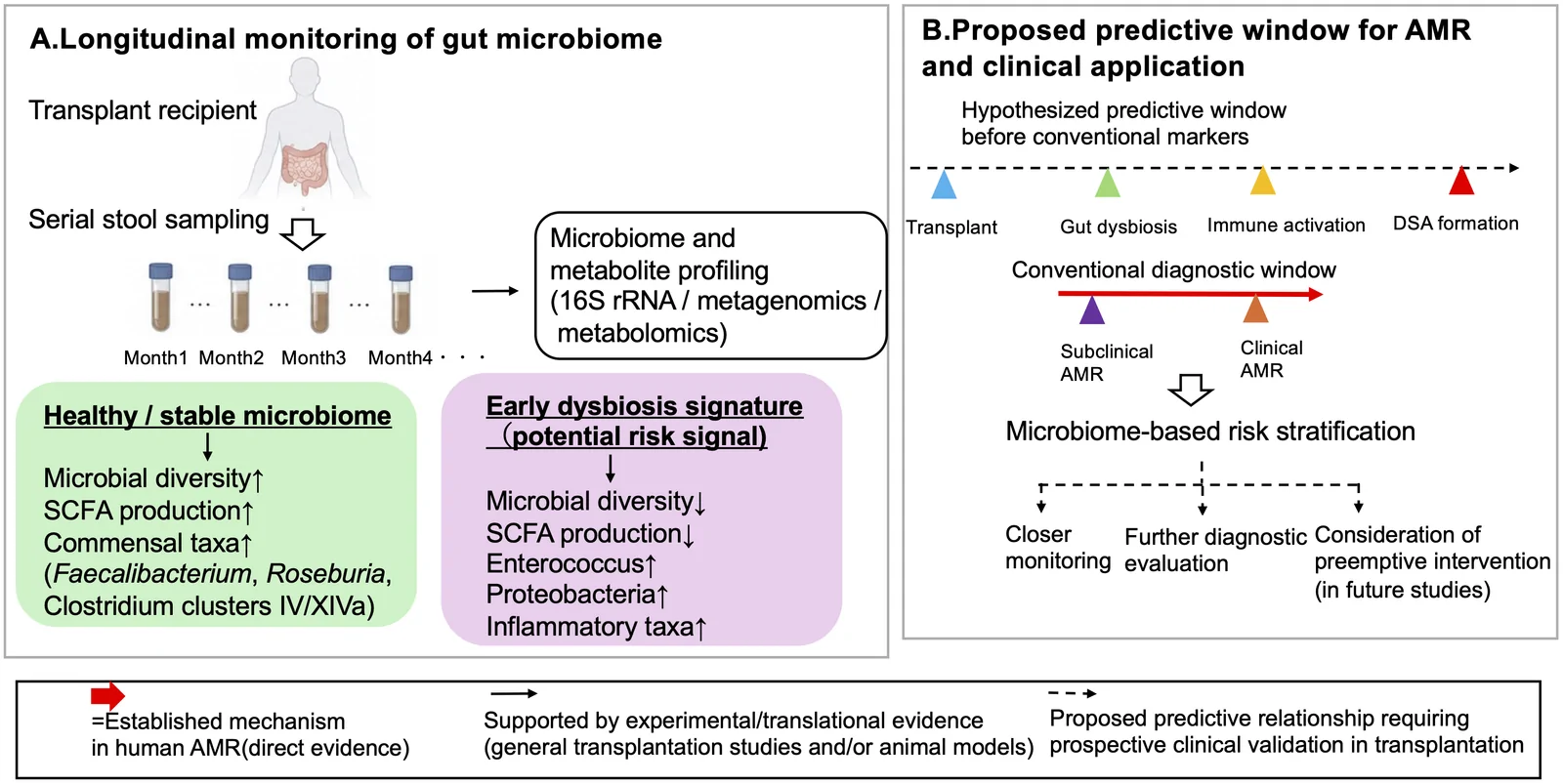
Clinical utility of longitudinal gut microbiome monitoring in predicting antibody-mediated rejection (AMR). **(A)** Serial profiling of the recipient gut microbiome may offer a non-invasive window for detecting immunological changes potentially preceding DSA formation, tissue injury, or clinical AMR becoming detectable (28, 30, 64, 70). Early dysbiosis patterns are typically marked by a reduction in microbial alpha diversity, depletion of key short-chain fatty acid (SCFA)-producing genera (Faecalibacterium, Roseburia, and Clostridium clusters IV/XIVa), and a corresponding overgrowth of inflammation-associated taxa such as Enterococcus and Proteobacteria (26–30, 68). This microbial imbalance is hypothesized to trigger barrier breakdown and microbe-associated molecular pattern (MAMP) translocation, providing the innate stimulus required for downstream T follicular helper (Tfh)- and B-cell activation to promote DSA generation and AMR pathogenesis (14, 17, 26–30, 38). **(B)** Because some studies suggest that these microbial and metabolite shifts may precede clinically apparent rejection, microbiome-based risk assessment may complement conventional diagnostic tools—including standard histology, Banff classification scoring, and donor-derived cell-free DNA (dd-cfDNA) assays (9–13, 28, 30, 64, 70). As indicated by the arrow designations, established clinical diagnostic phases are represented by solid lines, whereas the early microbiome-based risk window remains predictive. Integrating these metagenomic biomarkers into clinical risk stratification workflows is proposed as a potential framework to guide preemptive immunomodulatory interventions well before irreversible allograft damage occurs, although prospective clinical validation is strictly required to establish these proposed predictive relationships in transplantation. AMR, antibody-mediated rejection; dd-cfDNA, donor-derived cell-free DNA; DSA, donor-specific antibody; MAMP, microbe-associated molecular pattern; SCFA, short-chain fatty acid; Tfh, T follicular helper cell.

#### 3.1.1 Gut microbiome-based prediction and preclinical prediction of antibody-mediated rejection

Recent studies have demonstrated associations between gut microbiome alterations and transplant outcomes, including acute allograft rejection; however, AMR-specific human evidence remains limited. Transplant recipients experiencing rejection have been reported to exhibit reduced microbial diversity, depletion of SCFA-producing bacteria, and enrichment of pro-inflammatory taxa (26–30). Reduced abundances of butyrate-producing genera, including Faecalibacterium and Roseburia, may impair SCFA production and disrupt regulatory immune pathways mediated by Tregs and Bregs, thereby promoting inflammation (28, 30, 55–57, 68). Conversely, enrichment of Enterococcus and members of the phylum Proteobacteria has been associated with allograft rejection, chronic inflammation, and progressive deterioration of kidney allograft function (27–30). Crucially, a recent prospective study reported that distinct shifts in the gut microbiota and specific metabolite profiles are detectable prior to the clinical manifestation of acute rejection, further underscoring the clinical utility of preemptive risk stratification (69).

Accordingly, gut microbiome profiling may provide biomarkers for preclinical prediction and AMR risk stratification before irreversible graft injury occurs. As the gut microbiota is also amenable to therapeutic modulation, it represents a promising target for preventive interventions aimed at preserving long-term graft function (28, 30, 64, 70) (**Figure 4**).

### 3.2 Therapeutic modalities for gut microenvironment-mediated immunomodulation

#### 3.2.1 Conventional and emerging therapies for antibody-mediated rejection

Current treatment of AMR relies primarily on plasmapheresis, intravenous immunoglobulin (IVIG), and anti-CD20 monoclonal antibody therapy, such as rituximab (1–5, 71–73). However, LLPCs, the principal source of persistent DSA production, do not express CD20, limiting the efficacy of rituximab (2, 4, 8, 71, 73). Although IVIG exerts multiple immunomodulatory effects, including DSA neutralisation and inhibition of complement activation, it does not directly eliminate LLPCs and therefore has limited capacity to suppress sustained antibody production (2, 4, 71, 72).

To address these limitations, several novel therapeutic approaches have been investigated, including anti-CD38 monoclonal antibodies, interleukin-6 inhibitors, complement inhibitors, and IgG-degrading enzymes such as imlifidase. Nevertheless, no universally accepted treatment for AMR currently exists, and the condition remains a major unmet clinical challenge in transplantation (1–5, 7, 71, 73). Anti-CD38 therapy has attracted particular interest because of its ability to target LLPCs and suppress persistent DSA production (4, 5, 8, 74). However, because CD38 is also expressed on multiple immunoregulatory cell populations, including Bregs, activated Tregs, IgM mBCs, and natural killer cells, concerns remain regarding disruption of immune homeostasis and increased susceptibility to infection. The long-term efficacy and safety of these therapies thus remain to be fully evaluated (45, 51–54, 74).

#### 3.2.2 Impact of immunosuppressive therapy on the gut microenvironment

Furthermore, immunosuppressants prescribed for AMR prevention can induce gut dysbiosis, manifested by decreased microbial diversity and a loss of SCFA-producing taxa. These alterations may impair Treg- and Breg-mediated immune regulation and further disrupt immune homeostasis (16, 26, 28, 45, 52, 53, 55, 56, 75). Excessive immunosuppression also increases the risk of opportunistic infections, including Betapolyomavirus hominis 1 nephropathy and cytomegalovirus infection (76, 77). Thus, existing and novel therapeutic strategies must still balance effective immunosuppression with the preservation of protective immunity.

Although conventional immunosuppressive therapies are effective in controlling alloimmune responses, they may compromise immune homeostasis by disrupting the gut microbiota and reducing SCFA-producing bacteria (16, 26, 28, 53, 56). In contrast, the gut microenvironment provides insight into the dynamic interactions between the recipient immune system and the allograft, reflecting the balance between pathogenic humoral immunity and protective regulatory mechanisms (9, 14, 26, 28). Together, these limitations highlight the need for a conceptual shift from broad immunosuppression toward microbiome-targeted precision immunomodulation that preserves immune homeostasis while attenuating pathogenic alloimmune responses (9, 14, 26, 28) (**Figure 5**).

**Figure 5:**
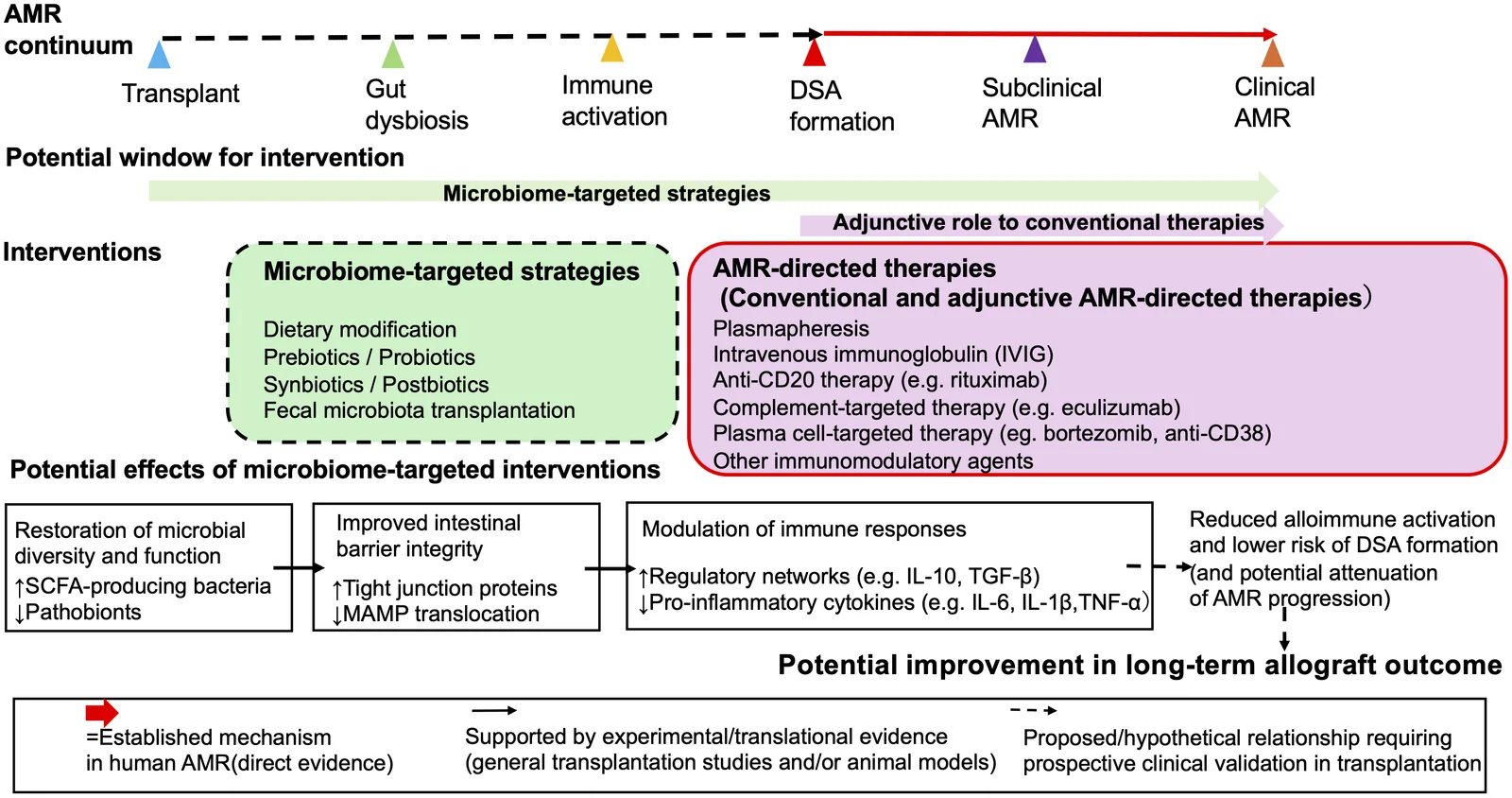
Conceptual shift from broad immunosuppression to microbiome-targeted precision immunomodulation. Standard AMR protocols rely heavily on broad-spectrum therapies including plasmapheresis, intravenous immunoglobulin (IVIG), anti-CD20 monoclonal antibodies, and complement inhibitors. While targeting specific humoral arms, these interventions often fail to fully eradicate long-lived plasma cells, disrupt host immune homeostasis, and carry substantial risks of toxicity and opportunistic infections (1–5, 7, 8, 52, 53, 71–74, 76, 77, 87). Aggressive immunosuppressive regimens further exacerbate gut dysbiosis by depleting beneficial short-chain fatty acid (SCFA)-producing taxa, thereby worsening systemic inflammation (16, 26, 28, 45, 52, 53, 55, 56, 75). Conversely, microbiome-targeted strategies—encompassing dietary changes, prebiotics, probiotics, synbiotics, postbiotics, and fecal microbiota transplantation (FMT)—are proposed to restore microbial diversity and reinforce barrier integrity (14, 28, 31–35). By mitigating microbe-associated molecular pattern (MAMP) translocation and reshaping systemic metabolism, these interventions are hypothesized to support regulatory networks (regulatory T cells [Tregs], regulatory B cells [Bregs], and IgM memory B cells) while attenuating the T follicular helper (Tfh)-germinal center axis responsible for plasma cell differentiation and donor-specific HLA antibody (DSA) synthesis (14, 17, 36, 45, 46, 51–57, 67, 75). As delineated by the visual framework, clinically established conventional protocols are enclosed in a solid panel, whereas microbiome-targeted strategies are demarcated by a dashed border to indicate their status as proposed preemptive modalities. Furthermore, the downstream causal pathways leading to reduced alloimmune activation and lower DSA risk are depicted with dashed arrows to explicitly represent speculative and hypothetical mechanisms requiring future prospective human clinical validation. This conceptual framework illustrates a potential transition toward more targeted immunomodulation across the AMR care continuum, but its clinical utility remains to be established. AMR, antibody-mediated rejection; Breg, regulatory B cell; DSA, donor-specific antibody; FMT, fecal microbiota transplantation; IVIG, intravenous immunoglobulin; MAMP, microbe-associated molecular pattern; SCFA, short-chain fatty acid; Tfh, T follicular helper cell; Treg, regulatory T cell.

#### 3.2.3 Postbiotics: from antibody-mediated rejection prevention to allograft protection

These observations have prompted increasing interest in microbiome-targeted therapies, including dietary interventions; prebiotics derived from dietary fiber and oligosaccharides; probiotics containing beneficial microorganisms such as Lactobacillus and Bifidobacterium species; synbiotics combining prebiotics and probiotics; and faecal microbiota transplantation (FMT), as adjunctive approaches to conventional immunosuppression (14, 28, 31–35).

Postbiotics—bioactive gut microbial metabolites such as SCFAs, indole derivatives, and secondary bile acids—represent potential therapeutic candidates in transplantation (14, 28, 31–34). Unlike probiotics, postbiotics do not require engraftment of live microorganisms and may therefore provide greater safety, stability, and reproducibility in immunosuppressed recipients (31–34).

Mechanistically, preclinical models suggest postbiotic metabolites could potentially modulate DSA generation by supporting Treg- and Breg-associated regulatory pathways while attenuating Tfh-cell responses and B-cell activation (51–53, 55–57). Postbiotic metabolites may also attenuate inflammatory pathways and potentially support endothelial protection. Such effects could theoretically contribute not only to reduced alloimmune activation before DSA formation but also to enhanced graft resilience after DSA development through mechanisms related to accommodation. However, direct evidence for these effects in DSA-positive transplant recipients remains limited (4, 5, 22–25).

Microbiome-targeted interventions may also promote expansion of SCFA-producing and other immunoregulatory bacterial taxa, supporting the maintenance of Tregs, Bregs, and IgM mBCs (14, 36, 45, 52–56). Modulating the gut microenvironment may therefore have potential as an adjunctive strategy for early risk assessment and, with further validation, for modulating alloimmune risk, but also potentially for supporting graft resilience in DSA-positive recipients.

Targeting the gut microenvironment is conceptualized as a promising strategy to move beyond empirical immunosuppression toward precision immunomodulation. By promoting immune regulation, potentially attenuating alloimmune responses, and enhancing allograft resilience, postbiotics represent a promising exploratory strategy for potentially improving long-term transplant outcomes. However, it must be emphasized that these biotics-based approaches remain largely within the domain of preclinical evaluation, and robust clinical evidence directly demonstrating their efficacy in human AMR cohorts is currently lacking.

Because postbiotics consist of defined bioactive molecules, they may provide greater standardization, reproducibility, and ease of manufacturing than live microbial products. Although clinical evidence in organ transplantation remains limited, further experimental and clinical studies are warranted to establish their efficacy, optimal formulations, and long-term safety. With further clinical validation, postbiotics may eventually contribute to precision immunomodulatory strategies in transplantation; however, their ability to prevent AMR, promote clinical tolerance, or preserve long-term allograft function remains to be established (**Table 2**).

**Table 2:** Comparison and current evidence of microbiome-based therapeutic approaches in transplantation.

Approach	Definition/components	Main immunological effects	Potential advantages/risks	Evidence in transplantation
Prebiotics/Probiotics/Synbiotics	Dietary fibers, live beneficial microorganisms, or their combinations	Modulation of microbial composition; potential enhancement of regulatory immune pathways	Relatively accessible; live microorganisms may carry risks of translocation or opportunistic infection	Emerging/limited clinical evidence
Postbiotics	Non-viable microbial components and/or bioactive metabolites, including SCFAs and indole derivatives	Modulation of Treg/Breg-associated pathways; anti-inflammatory and barrier-supporting effects	Defined composition; greater stability and reproducibility; lower infectious risk than live microbial approaches	Primarily preclinical/exploratory in transplantation; clinical efficacy for AMR not established
FMT	Transfer of processed fecal microbiota from a screened healthy donor	Broad restoration of microbial ecosystem and metabolic functions; potential restoration of immune homeostasis	Broad ecosystem restoration; risks of donor-derived infection, variable composition, and difficult standardization	Clinical use established mainly for recurrent *C. difficile* infection; transplantation-specific immunomodulatory application remains investigational

#### 3.2.4 Faecal microbiota transplantation

Beyond metabolite-based interventions, FMT represents a comprehensive microbiome-targeted intervention that restores both the composition and functional capacity of the intestinal ecosystem. Unlike prebiotics, probiotics, and postbiotics, which target specific microbial components or metabolites, FMT aims to reconstruct the entire gut microbial community, thereby restoring microbial diversity, intestinal barrier integrity, and microbiota-derived metabolite production (28, 35, 78–81).

Experimental evidence suggests that FMT may promote expansion of SCFA-producing bacteria, restore microbial homeostasis, reinforce intestinal barrier function, and modulate both innate and adaptive immune responses. These effects may contribute to suppression of alloimmune activation by promoting regulatory immune cell populations, reducing systemic inflammation, and re-establishing immune homeostasis (35, 78, 79, 81).

Importantly, direct transplantation-specific evidence has been reported in a murine cardiac allograft model, in which microbiota transfer from distinct donor environments resulted in different graft outcomes. Enrichment of Bifidobacterium pseudolongum was associated with prolonged allograft survival and reduced graft inflammation and fibrosis, and administration of this bacterial species alone reproduced several immunoregulatory effects (59). These findings suggest that both microbial community composition and specific bacterial taxa can modulate alloimmune responses.

Although clinical experience with FMT in solid organ transplantation remains limited and confined mostly to infectious complications like recurrent Clostridioides difficile, preliminary studies have suggested potential benefits in correcting dysbiosis and restoring immune regulation in transplant recipients. However, important challenges remain, including donor selection, pathogen screening, standardization of transplantation protocols, and long-term safety, particularly in immunosuppressed patients (35, 80, 82). Further preclinical and clinical studies are therefore warranted to establish whether FMT can be safely translated into a therapeutic strategy for preventing antibody-mediated rejection, promoting immune tolerance, and preserving long-term allograft function. However, clinical translation of FMT in immunosuppressed transplant recipients requires extreme caution due to the risk of donor-derived bacteremia and severe infectious complications, as well as the inherent challenges in standardizing live donor-derived products (52, 53, 67, 74) (**Table 2**). A comprehensive comparative analysis of these microbiome-targeted therapeutic approaches, with a specific focus on the safety profiles and immunomodulatory mechanisms of postbiotics versus other modalities, is summarized in **Table 2**.

## 4 Challenges in the clinical translation of gut microenvironment-based approaches

### 4.1 Current challenges and future perspectives in diagnosis

Several challenges must be addressed before gut microenvironment-based approaches can be translated into routine clinical practice (28, 30, 62–64, 70).However, current technologies, including 16S ribosomal RNA gene sequencing and shotgun metagenomic sequencing, remain limited by high costs, prolonged turnaround times, analytical complexity, and a lack of standardization across institutions. Furthermore, the temporal dynamics of the gut microenvironment and post-transplant immune responses pose significant challenges to real-time monitoring. Addressing these challenges will require integrated diagnostic workflows combining microbiome, metabolomic, and immune profiling approaches (28, 62, 64, 70). Equally important will be the development of rapid, cost-effective, and scalable diagnostic platforms capable of monitoring gut microbial status and predicting AMR risk in real time (28, 62, 64). Standardization of sampling procedures, analytical methods, and timing of assessment will also be essential, given the substantial influence of diet, antimicrobial therapy, immunosuppressive agents, and infection-related complications on the gut microenvironment (26, 28). Identifying AMR-specific microbial and metabolite signatures, developing clinically applicable risk-scoring systems, and implementing AI-assisted multi-omics analyses remain key priorities for improving diagnostic accuracy and predictive performance (30, 62–64, 70).

#### 4.1.1 Artificial intelligence-driven multimodal integration for precision transplantation

Future advances in transplant immune monitoring are likely to depend on integrating complementary biological, imaging, and clinical information (28, 30, 62–65). Complementing multi-omics approaches, AI-assisted image analysis—including automated Gram stain microscopy and histopathological evaluation—provides valuable phenotypic insights alongside metagenomic, metabolomic, and transcriptomic profiling. AI-driven multimodal integration of these datasets with clinical variables enables comprehensive characterization of host–microbiome interactions, allowing identification of microbial metabolic shifts (such as reduced butyrate or tryptophan metabolites) associated with endothelial activation, complement C4d deposition, and Treg/Tfh imbalance during rejection. Machine-learning models, including Random Forest, XGBoost, and deep neural networks, may therefore improve early AMR prediction, treatment-response assessment, and personalized immunomodulatory strategies (28, 30, 62–65). Ultimately, these approaches could support individualized precision transplantation by tailoring immune monitoring and therapeutic interventions to each patient’s unique gut microenvironment and immunological profile (28, 30, 62–65).

However, several clinical and translational challenges must be resolved before these AI-driven models can be routinely applied in practice. At present, these computational frameworks remain strictly in the exploratory or discovery phase. First, current transplantation datasets are often limited by small cohort sizes, increasing the risk of model overfitting and reducing generalizability (83). Second, the inherent “black-box” nature of complex machine-learning algorithms (particularly deep neural networks and ensemble tree models) limits clinical interpretability, making it difficult for clinicians to understand the underlying biological mechanisms driving predictions (84). Third, substantial inter-center variation in sample collection, sequencing platforms, and data processing presents a major barrier to multi-center data standardization (82). Overcoming these limitations will require large-scale, multi-center prospective validation and the development of explainable AI (XAI) frameworks.

### 4.2 Current challenges and future perspectives in therapeutic management

The gut microenvironment represents a potentially modifiable therapeutic target through interventions such as prebiotics, probiotics, synbiotics, postbiotics, and FMT (14, 31–35). However, the optimal microbial taxa, metabolite profiles, treatment strategies, timing of intervention, and long-term safety remain to be established. Caution is required in immunosuppressed transplant recipients, in whom the risks of infection and unintended immune activation must be carefully balanced against potential therapeutic benefits (28, 35). Furthermore, while expanding regulatory pathways—such as FoxP3+ Tregs—is beneficial for preventing allograft rejection, an excessive or uncontrolled increase in Treg populations may inadvertently lead to over-immunosuppression (14, 21). This raises a critical concern regarding the impairment of protective antimicrobial immunity, potentially increasing the risk of chronic infections, opportunistic pathogen proliferation, or the reactivation of latent viruses, such as cytomegalovirus or BK polyomavirus (76, 77). Therefore, balancing the promotion of transplant tolerance with the preservation of anti-infective immunity remains a major translational challenge.

It remains to be determined whether restoring the gut microenvironment can reliably re-establish regulatory immune networks involving Tregs, Bregs, and IgM mBCs. Consequently, its ultimate efficacy in maintaining immune homeostasis, preventing AMR, and enhancing treatment responsiveness requires clarification. Addressing these questions will be critical for translating microbiome-based interventions into clinical practice (14, 36, 45, 52, 55, 56, 66, 67). As these challenges are addressed, integration of gut microbiome profiling with immune monitoring may enable personalized immunosuppressive strategies, providing the foundation for precision immunomodulation in AMR management.

#### 4.2.1 Gut microbiome-guided personalized immunomodulation

Current immunosuppressive therapy largely relies on empirical treatment strategies, with drug selection and dose adjustment based primarily on clinical experience, therapeutic drug monitoring, and conventional laboratory parameters. However, substantial interindividual variability remains in treatment efficacy, adverse events, and susceptibility to infection, highlighting the need for more personalized therapeutic approaches (13, 85, 86).

Beyond disease prediction, AI-driven integration of gut microbiome profiles with multi-omics, pharmacokinetic, pharmacodynamic, and clinical data may facilitate personalized therapeutic decision-making (13, 62, 85, 86).

Such approaches could facilitate patient-specific selection and optimisation of immunosuppressive agents, including calcineurin inhibitors, mycophenolate, costimulation blockade, anti-CD38 antibodies, interleukin-6 inhibitors, and complement inhibitors, thereby advancing precision pharmacology in transplantation (1–5, 71, 74, 85–87).

Microbiome-guided therapeutic stratification may optimize immunosuppressive dosing, minimize treatment-related toxicity, and improve long-term graft outcomes. Future integration with AI-driven decision support and companion diagnostics may further advance precision immunomodulation in transplantation (13, 62, 85, 86).

## 5 Conclusion

The gut microenvironment represents a promising source of biomarkers and therapeutic targets that reflect the balance between humoral immune responses and immune regulatory mechanisms involved in AMR. Analysis of the gut microbiota and microbiota-derived metabolites may facilitate preclinical prediction, risk stratification, and personalized therapeutic decision-making in transplant recipients. Gut microenvironment modulation—using biotics or FMT—is conceptualized as a promising strategy to shift AMR management from conventional immunosuppression toward precision immunomodulation by potentially expanding FoxP3+ Tregs and IL-10-producing Bregs while theoretically attenuating Tfh cell-mediated plasma cell differentiation.

Future integration of microbiome profiling, metabolomics, and AI-driven multi-omics analyses with clinical and histopathological data may enable dynamic, individualized immune monitoring capable of identifying patients at risk before irreversible graft injury develops. Such approaches could transform transplantation from reactive treatment of established rejection to proactive precision immunomodulation through earlier diagnosis, improved risk stratification, and timely intervention. Beyond transplantation, these technologies may also advance the understanding and management of a broad spectrum of immune-mediated diseases, including autoimmune disorders, allergic diseases, infectious diseases, and cancer. Prospective, multicenter validation studies will be essential to establish standardized protocols and confirm the clinical utility of gut microenvironment-guided diagnostic and therapeutic strategies in organ transplantation.
